# Letter from the Editor in Chief

**DOI:** 10.19102/icrm.2024.15026

**Published:** 2024-02-15

**Authors:** Moussa Mansour


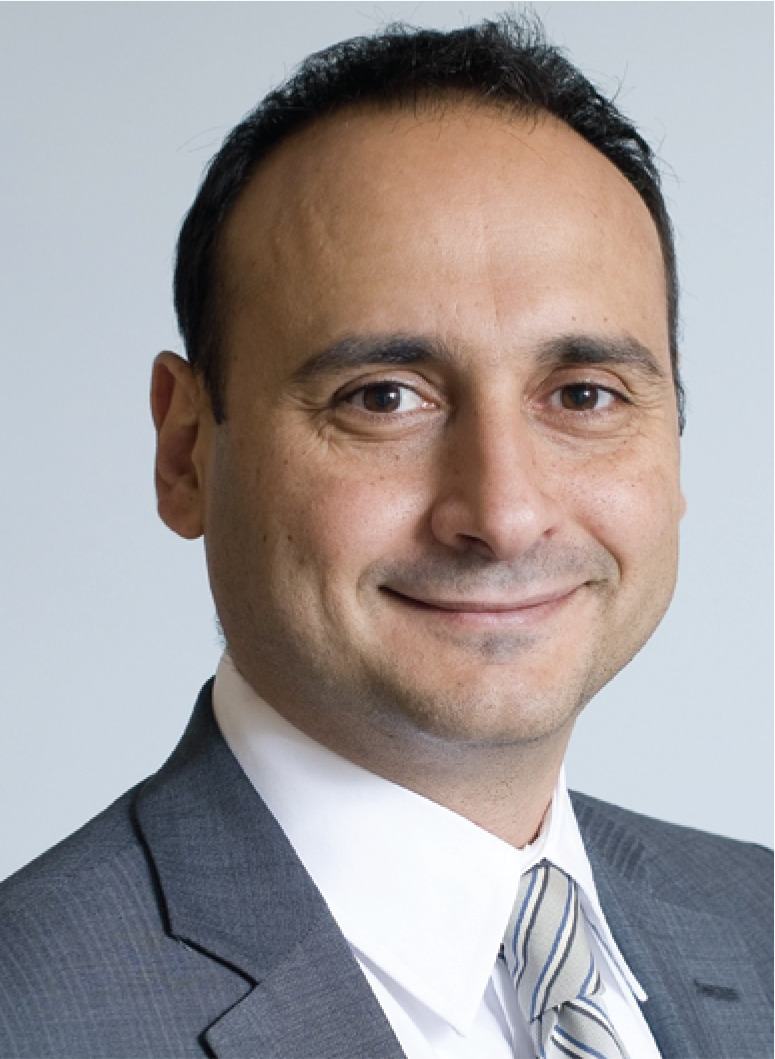


Dear readers,

The 29th annual AF Symposium was held in Boston on February 1–3, 2024. The symposium hosted more than 1,300 attendees from all over the world. Eighty-five national and international faculty speakers presented their most recent science in the form of lectures and live and recorded cases of ablation for atrial fibrillation (AF) and left atrial closure. There were also 65 poster abstracts and one oral presentation for the Best Abstract Award. Numerous novel technologies were presented during the Spotlight sessions, and new devices were displayed by health care companies in the exhibit hall. One of the highlights of the AF Symposium was the late-breaking clinical trial (LBCT) sessions, which contained 10 presentations given over 2 days. While all 10 presentations are important and likely to have significant impacts on clinical practice, I will highlight three here that are related to the safety of pulsed-field ablation (PFA), which were all simultaneously published in major electrophysiology journals.

The first LBCT of note was presented by Dr. Andrea Natale and published simultaneously in *JACC: Clinical Electrophysiology*. It is titled “Acute Kidney Injury Resulting from Hemoglobinuria Following Pulsed-field Ablation in Atrial Fibrillation: Is It Preventable?.”^1^ Hemolysis has been recognized as a potential complication of PFA. Its occurrence is low and results from red blood cell lysis when subjected to a voltage pulsation. The released free hemoglobin can cause tubule-barrier deregulation and oxidative cell damage and subsequent acute kidney injury. The study enrolled two groups of patients, including 28 patients who did not receive post-ablation hydration and 75 patients who received ≥2 L of normal saline after ablation. The authors found that the rate and severity of the increase in serum creatine was related to the number of ablation applications. Moreover, after ablation, there was an increase in serum creatinine of 0.68 ± 0.76 mg/dL in the first group compared to no change in the group that received hydration. The authors concluded that post-procedure hydration can prevent acute kidney injury after PFA.

The second LBCT of note was published simultaneously in *Europace* and titled “Pulmonary Vein Narrowing after Pulsed Field vs. Thermal Ablation: A Prespecified Secondary Endpoint of the Randomized ADVENT Trial.”^2^ The Randomized Controlled Trial for Pulsed Field Ablation versus Standard of Care Ablation for Paroxysmal Atrial Fibrillation (ADVENT) study demonstrated that PFA was non-inferior to conventional thermal ablation with respect to the primary endpoint of freedom from atrial arrhythmias.^3^ This study reported the result of one of the secondary endpoints of ADVENT, which consisted of a prespecified comparison of the change in pulmonary vein (PV) ostial dimensions between the PFA and thermal groups. The endpoint of the study was met, demonstrating that PFA is superior to thermal ablation with regard to its effect on PV dimension. Specifically, the change in PV cross-sectional area was less with PFA (−0.9%) than with thermal ablation (−12%, posterior probability > 0.999), and this was primarily driven by the radiofrequency subcohort (−19.5%) vs. cryoballoon subcohort (−3.3%). The diameters of almost half of all PFA-treated PVs did not decrease, but the majority (80%) of radiofrequency-treated PVs decreased.

The third LBCT, presented by Dr. Yury Malyshev and published simultaneously in *JACC: Clinical Electrophysiology*, was titled “Long-term Effects of Pulsed Field Ablation on Coronary Arteries.”^4^ It has been established now that PFA can cause coronary artery spasm if ablation is performed near a coronary artery. While all previous studies on this topic have described the transient effect of PFA on coronary arteries and how it can be prevented using intravenous nitroglycerine, this study was the first to analyze the potential chronic impact. Seventy-seven patients who developed angiography-proven coronary spam during ablation of the mitral and tricuspid isthmuses were included in this study and underwent repeat coronary angiography more than 6 months after the index procedure. Coronary irregularity or luminal narrowing was not seen at the site of prior spasm in any patient. The authors concluded that PFA-induced vasospasm leads to neither chronic vascular changes nor angiographically significant coronary stenosis.

In addition to these three studies, there was also a notable lecture presentation given by Dr. Vivek Reddy on the results of MANIFEST-17K. This was a registry study of 17,7642 patients who underwent ablation at European centers using a pentaspline PFA catheter, and no evidence of atrio-esophageal fistula, PV stenosis, or persistent phrenic nerve paralysis was documented in this large group pf patients.

Put together, all the studies described above provide strong evidence that PFA is by far a safer technology than thermal ablation for the treatment of AF.

Thank you for reading this issue of *The Journal of Innovations in Cardiac Rhythm Management*.


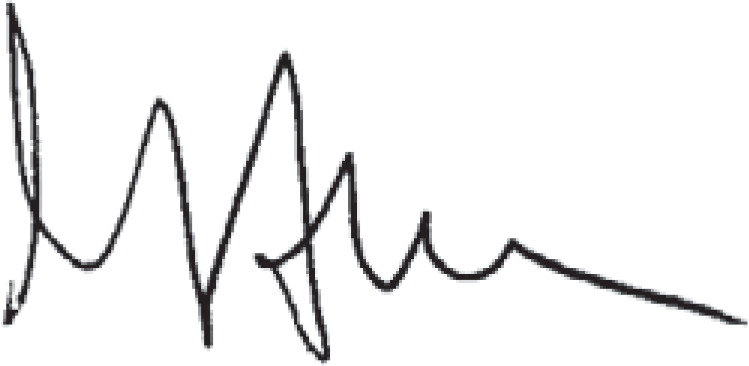


Best personal regards,

Moussa Mansour, md, fhrs, facc

Editor in Chief

*The Journal of Innovations in Cardiac Rhythm Management*

MMansour@InnovationsInCRM.com

Director, Atrial Fibrillation Program

Jeremy Ruskin and Dan Starks Endowed Chair in Cardiology

Massachusetts General Hospital

Boston, MA 02114
